# Insights into an NEk2 inhibitory profile of nitidine chloride by molecular docking and biological evaluation

**DOI:** 10.1186/s13065-022-00870-6

**Published:** 2022-10-09

**Authors:** Danni Li, Jiahao Lu, Qiying Zhang, Yuzhu Zhou, Long Li, Hua Zhu, Tong Li

**Affiliations:** 1 School of Chemistry and Chemical Engineering, Guangxi Key Laboratory for Polysaccharide Materials and Modifications, Guangxi Minzu University, No.158, Da Xue Xi street, Xixiangtang District, Nanning, 530006 Guangxi China; 2 College of Pharmacy, Guangxi University for Chinese Medicine, No.13, Wu He street, Qingxiu District, Nanning, 530200 Guangxi China

**Keywords:** Nitidine chloride nanoparticle, Li-7 hepatocellular carcinoma cells, Nitidine chloride, MOE molecular docking, NEK2 protein

## Abstract

Deregulation of NEK2(NIMA-related serine/threonine 2) confers chemotherapeutic resistance to apoptosis and is closely correlated with poor prognosis in hepatocellular carcinoma (HCC). Here, we find that nanoparticles are prepared through hemisynthesis from natural nitidine chloride (NC) with enhanced antitumor activity. Nitidine chloride nanoparticle (TPGS-FA/NC) treatment show good therapy effect in Li-7 hepatocellular carcinoma cells. Additionally, molecular docking technologies are aimed at NEK2 protein (PDB ID: 6SGD) to analyze the detailed binding interactions with the potent target. NC participates in interactions with Asp159 residue. These studies advance the understanding of the modification of nitidine chloride substituent and provide useful drug design information for liver cancer treatment.

## Introduction

**Fig. 1:**
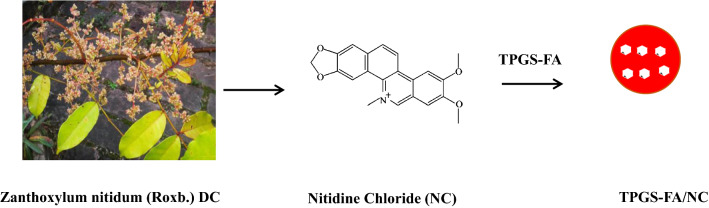
Chemical structure of nitidine chloride (NC)

The population of cancer incidence and deaths in 2016 was higher than in previous years [1–3]. Hepatocellular carcinoma (HCC) is the second leading cause of cancer-related death [4]. HCC remains recurrence rate for more than 70% patients at five years. The clinical drugs prove limited therapeutic efficacies in liver cancer therapy [5–7]. There is an urgent to find more potential drugs. Tumor initiating cells (TICs) show stepwise progression during the cancer formation in HCC. CD133^+^EpCAM^+^ phenotype is a distinguishing feature of TICs in HCC. Furthermore, the carcinogenesis role of aquaporin 3 (AQP3) has been identified mechanism of the stem cell and TICs by high expression levels of CD133 in HCC patients [8]. Considering the critical role of TICs in apoptosis resistance by hepatocellular carcinoma cells, targeted drugs designed to suppress anti-apoptotic proteins need to be developed [9]. In our previous study, we have designed and synthesized nitidine chloride nanoparticles (Fig. 1) by folic acid modifying D-α-tocopheryl polyethylene glycol 1000 succinate (TPGS-FA) as a promising carrier for controlled delivery of the drug. Characterizations analysis of TPGS-FA/NC demonstrate the properties of good water solubility and favorable size [10]. The novelty of our work comes from the fact that the benzophenanthridine alkaloid as potential selective drug for inhibition of hepatocellular carcinoma cells in proliferation processes. A non-catalytic cysteine (Cys22) residue is considered as characteristic catalytic domain in NEk2 protein family [11]. However, the presence of H-donor, particularly the aspartic (Asp159) residue, is significant for suggesting different binding domain of NEk2. Our findings provide insights into TPGS-FA/NC-NEk2 docking as a potent drug discovery strategy that can overcome apoptotic resistance in liver cancer.

## Materials and methods

TPGS-FA/NC was synthesized and characterized as previously described [10]. Li-7 cell was purchased from Shanghai Cell Bank (Shanghai Institute for Biological Science, Chinese Academy of Science, Shanghai, China). DMEM mediawas purchased from Life Technologies (AB & Invitrogen Gibco, Suzhou, China). Fetal bovine serum (FBS) was purchased from Gemini (Gemini Calabasas, CA, source: fetal bovine, USA). Recombinant human bFGF (bFGF), recombinant human 1epidermal growth factor (EGF) and MTT were purchased from Beijing Solarbio Science & Technology Co. Ltd (Solarbio, Beijing, China). B27 (× 50) was purchased from Thermofish Scientific (Thermofish,waltham, USA). DMEM/F-12, Insulin-Transferrin-Selenium (ITS × 100) and L-glutamine (× 100) were purchased from procell Science&Technology Co.Ltd (Procell, Wuhan, China). NEK2, AQP3, EpCAM were purchased from Beijing Solarbio Science & Technology Co. Ltd. ( Solarbio, Beijing, China). CD133 was provided for the Proteintech Science&Technology Co.Ltd.(Proteintech, Suzhou, China). Normal goat serum was purchased from Beijing Solarbio Science & Technology Co., Ltd. ( Solarbio, Beijing, source: normal goat serum liquid, China). DAPI and reactive Oxygen Species (ROS) detection assay Kit were obtained from Shanghai Beyotime Biotechnology Co.Ltd. (Beyotime, Shanghai, China). Ifluor^TM^ 647 phalloidin was purchased from Yeasen Biotechnology Co., Ltd (Yeasen, Shanghai, China). 5-fluorouracil (5-Fu) was purchased from Med Chem Express (MCE, Monmouth Junction, NJ, USA). Crystal violet was purchased from the Beijing Solarbio Science & Technology Co.Ltd. (Solarbio, Beijing, China).

### Instruments and apparatus

Li-7 cells were cultured at 37 °C, 5% CO2 cell incubator(Thermo Fisher,USA). The absorbance (Am) was measured at 490 nm using a microplate reader (Thermo Fisher, USA). The Morphology of cells was observed on a inverted phase contrast microscope (Olympus, China). The cell imaging was performed using Leica confocal microscope (Leica SP8 Corporation, Germany). Mean fluorescence intensity was was determined on the flow cytometer (BD Accuri ^®^ C6 PLUS Bio-sciences, USA). The immunohistochemistry measurements were assayed by the bioluminescence microscope(Olympus BX43, Japan).

### Synthesis of TPGS-FA/NC nanoparticles

The TPGS.NH_2_ dendrimers were conjugated with FA as described in our previous described [10]. Following with our previously published procedure, encapsulation of NC within TPGS-FA dendrimers was obtained and characterized [10].

### Procedure for molecular docking studies

Finally, to obtain a 3D view of NEk2/NC complex, we performed in nitidine chloride molecular docking studies in the active site of NEk2, using software MOE 2014 [12]. The Site-Finder function of the MOE software was performed to confirm that the maximum cavity was the binding pocket of the 6SGD protein. The generalized-born volume integral/weighted surface area (GBVI/WSA) dG scoring (default for Rescoring 2) was set. Rescoring 2 was performed to calculate the binding free energy of ligands and receptors via AMBER99 and MMFF94x force field models followed by the proper and favorable docking arrangement. The waters and ligands were removed from 6SGD protein, 6SGD and NC were protonated and energy was minimized by AMBER99 and MMFF94x force field models. Those distribution of RMSD and binding energy were analyzed using molecular docking software. The structure of top rescoring 2 value was added to binding pocket. Finally, NEK2 protein–NC interactions were analyzed.

### Biological evaluation

#### Cell culture

Li-7 cells were cultured in DMEM medium supplemented with 10% fetal bovine serum, 100 U/mL penicillin and 50 mg/mL streptomycin at 37 °C in a humidified 5% CO_2_ incubator.

#### Cytotoxicity assay

Li-7 cells (2–3 × 10^3^cells/well) were seeded in 96-well white plates and incubated with serial dilutions of TPGS-FA/NC including 5-Fu, NC or vehicle (1%DMSO) to a final volume of 200μL. Cell viability was measured at 24, 48 and 72 h by addition of MTT Assay reagents according to the manufacturer’s protocol (Solarbio). Fixed cells with 4% formaldehyde were stained with crystal violet according to the standard procedure.

#### Tumor sphere formation assay

Briefly, primary sphere cells were obtained by culturing Li-7 cells in sphere-forming conditioned Dulbecco’s modified Eagle’s medium/Ham’s F12 medium (DMEM/F12) supplemented with FGF (20 ng/mL), EGF (20 ng/mL), B27 (1 ×), ITS (1 ×), and L-glutamine (1 ×) in 6-well ultra-low attachment plates. The primary sphere cells (1 × 10^3^cells/well) were incubated with or without TPGS-FA/NC for 7d. The second and third passages of the cells were grown for 7 days in the absence of TPGS-FA/NC. Cell sphere counts (> 50 μm in diameter) were determined on a phase contrast microscope as previously described [9].

#### Confocal microscopy imaging

Firstly, Li-7 cells were seeded in glass slide and incubated overnight at 37 °C. Cells were incubated with Rhodamine B isothiocyanate 540 loaded nanoparticles (final concentration of 100 nM) for 4 h at 37 °C. Cells were washed twice with PBS and fixed with 4% formaldehyde, followed by treatment with 0.1% Triton X-100 for 5 min. The cell cytoskeleton was incubated with iFluor TM 647 phalloidin iFluor™ for 30 min at room temperature. After washing with PBS, cells were mounted with ProLong @ Gold Antifade Reagent containing DAPI for cell nucleus staining. Samples were collected for observation and analysis.

#### Reactive oxygen species (ROS) level

Negative group: Single cell suspensions were prepared in HMEM (1 mL). Sample group: Single cell suspensions were prepared in HMEM (1 mL) and DCFH-Da (1 μL) (final concentration of 10 μM). Samples were mixed and incubated for 30 min away from light. The supernatants were isolated by centrifugation at 1500 × rpm spin for 5 min. After washing in HMEM (1 mL), supernatants were isolated by centrifugation at 1500 × rpm spin for 5 min. Finally, Cells were resuspended in PBS (200μL), mean fluorescence intensities were detected by a BD Accuri ^®^ C6 PLUS flow cytometer.

#### Immunohistochemistry (IHC)

Immunohistochemical staining samples were conducted to standard procedure. Briefly, cells were seed in glass slide and incubated with 40 μg/mL TPGS-FA/NC. After 48 h, fixed cells were soaked in distilled water for 5 min twice. Hydrogen peroxide (3%)was performed to block the endogenous peroxidase activity and normal goat serum was used to block non-specific staining for 30 min at 37 °C. Primary antibodies were used to detect the following proteins:AQP3, CD133, EPCAM, NEK2 overnight at 37 °C. Cells were incubated with biotinylated goat anti-rabbit IgG secondary antibody followed by staining with DAB reagent and counterstaining with hematoxylin. To evaluate IHC staining of proteins in the nuclear and cytoplasmic regions, protein mean densities were detected by a bioluminescence microscope.

#### Statistical analysis

Statistical tests were generated in GraphPad Prism 6.0. Data are presented as means ± SD except where noted. Statistical comparisons between two groups were performed using an unpaired t-test. Statistically significant differences were denoted as *p < 0.05, **p < 0.01, and ***p < 0.001.

## Results and discussion

### Molecular docking

**Fig. 2:**
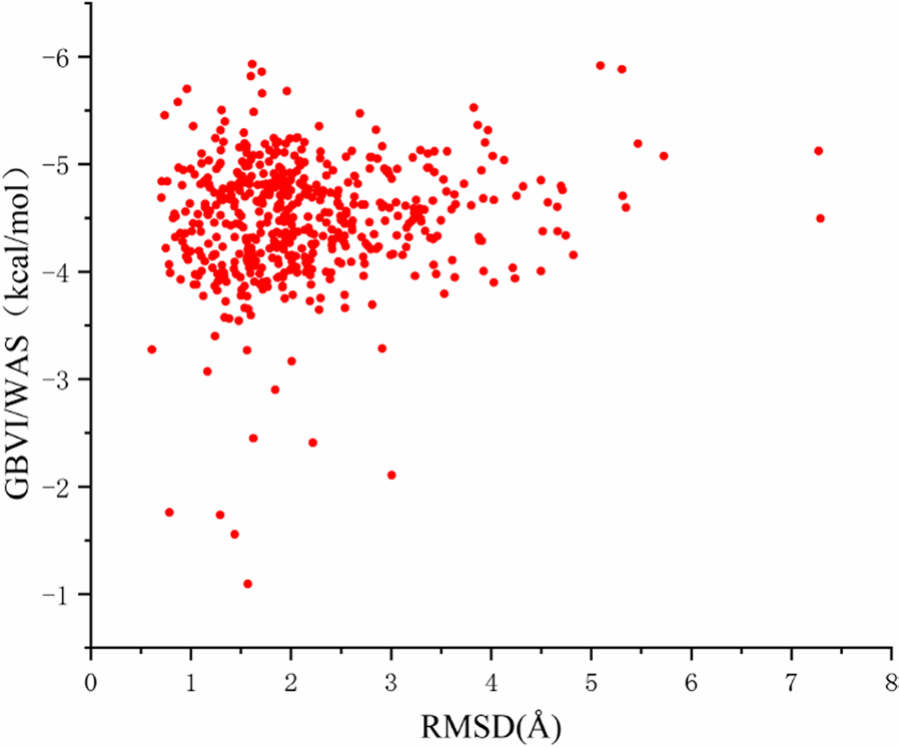
NC multi-reconstruction image and RMSD coordinate diagram

**Fig. 3:**
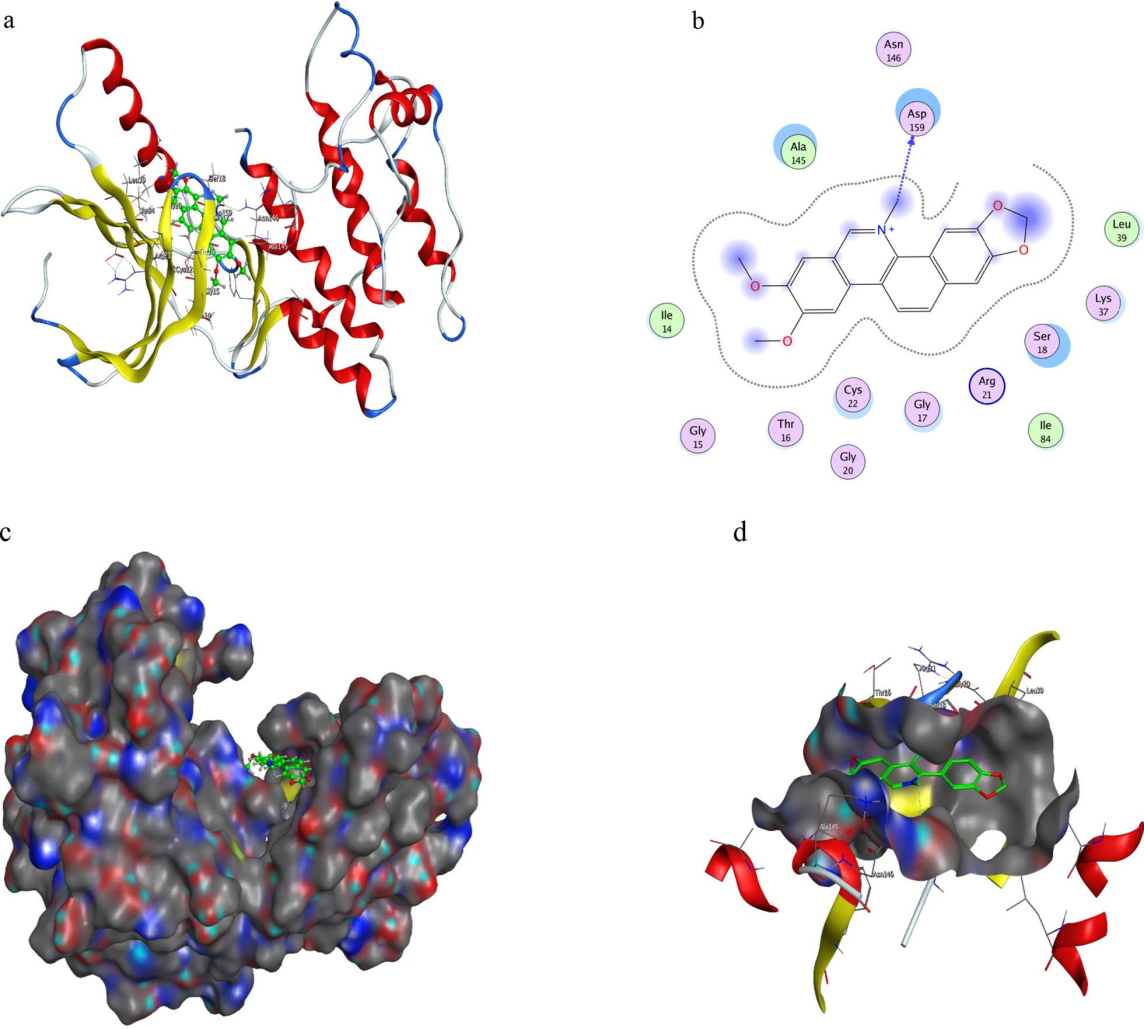
**a** Binding sites of NEK2 **b**: Docking pose interaction diagram for NC in NEK2 gorge **c**: Docked pose of NC into the binding sites of NEK2, (NC is shown in colored spheres) **d**: zoomed-in view showing the interactions of NC with the residues of NEK2 (NC is shown in spheres)

The coordinates of the protein structure were obtained from Protein Data Bank by selecting the structure with accession code 6SGD. The 573 different conformations were found through molecular docking results and then relationships between various configurations and RMSD were analyzed (Fig. 2). Furthermore, molecular docking investigations into the active site of NEK2 yielded well-clustered solutions for nitidine chloride binding modalities and produced a binding affinity in the range of − 3.5 to − 5.5 kcal/mol. RMSD values of the constructions were concentrated in the range of 1 to 3.5 Å. The optimal configuration and GBVI/WSA binding energy of NEK2 was − 5.926 kcal/mol. Especially, one of the quaternary amines sat well in the binding site, which N atom established an H-donor with Asp159. The binding free energy of H bond was found to be − 0.9 kcal/mol and atomic distance was 3.25 Å. Additionally, binding free energy of arene-H bond was found to be -0.7 kcal/mol and atomic distance was 3.87 Å. Thirteen residues with Ala145,Asn146,Ile14,Gly15,Thr16,Gly20,Cys22,Gly17,Arg21,Ile84,Ser18,Lys37, or Leu39 showed poor interactions. According to the molecular docking studies, the methyl on the N atom was replaced with H atom to increase the hydrophilicity of NC,N atom interaction with Asp159 was enhanced. Taken together, hydrophobic substituents on the naphthalene ring were beneficial to increase the binding action of NC and amino acids (Fig. 3). Thus, our data is consistent with the interaction of NEK2 with TPGS-FA/NC as determinant of the downregulation of protein expression levels (Fig. 9).

### TPGS-FA/NC inhibits Li cells proliferation and colony formation

**Fig. 4:**
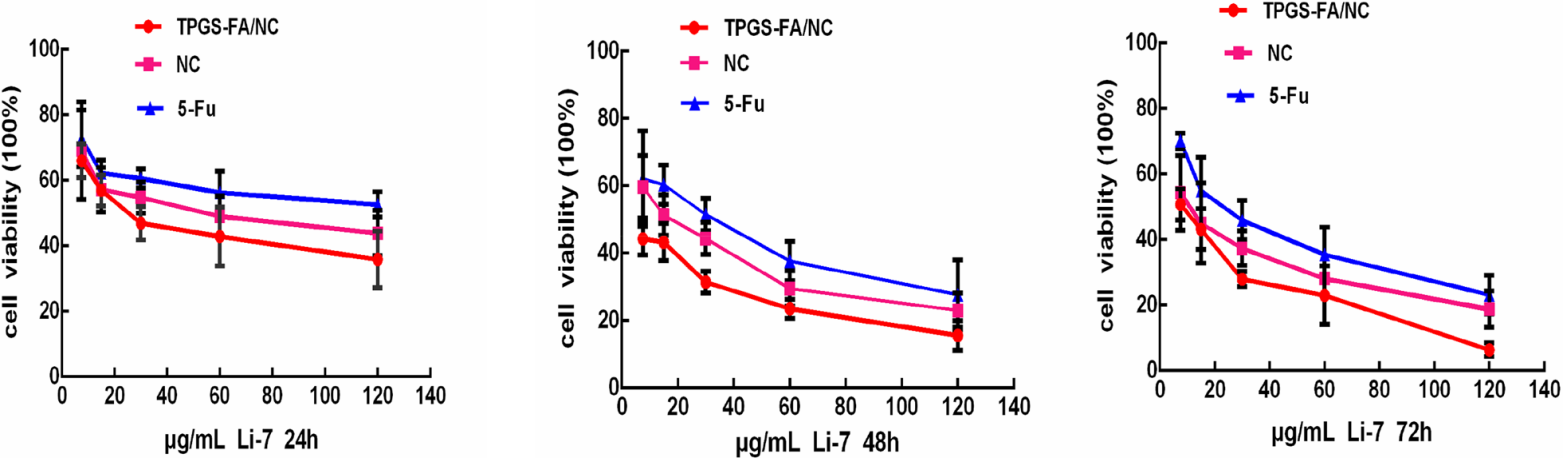
Cell viability at 24,48 and 72 h in Li-7 cell treated with TPGS-FA/NC, NC and 5-Fu

**Fig. 5:**
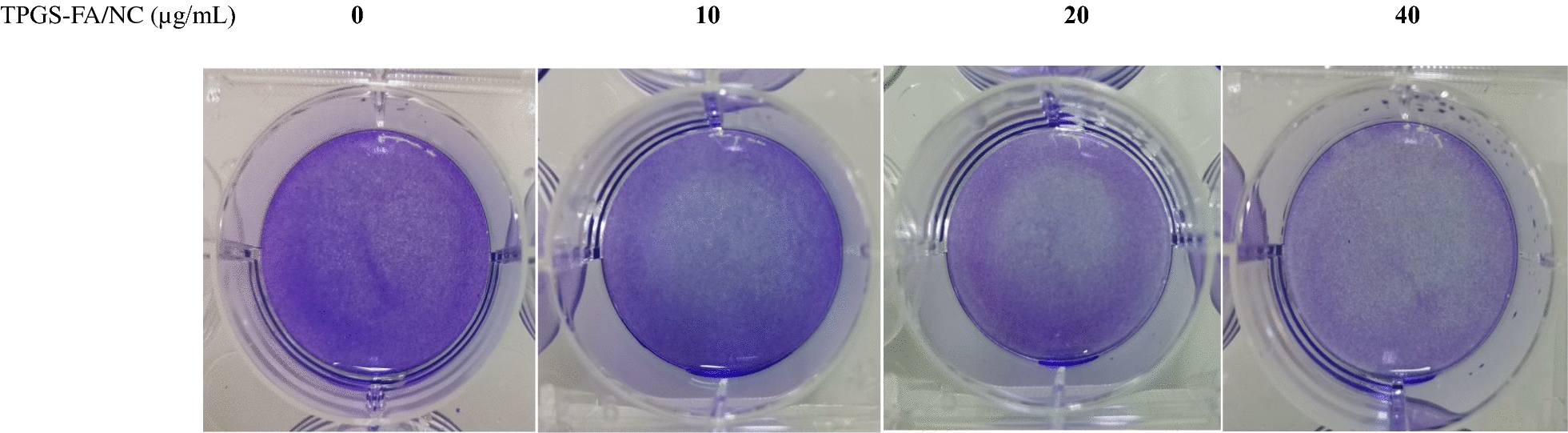
Crystal violet staining of Li-7 cells which was treated with TPGS-FA/NC for 48 h

**Fig. 6:**
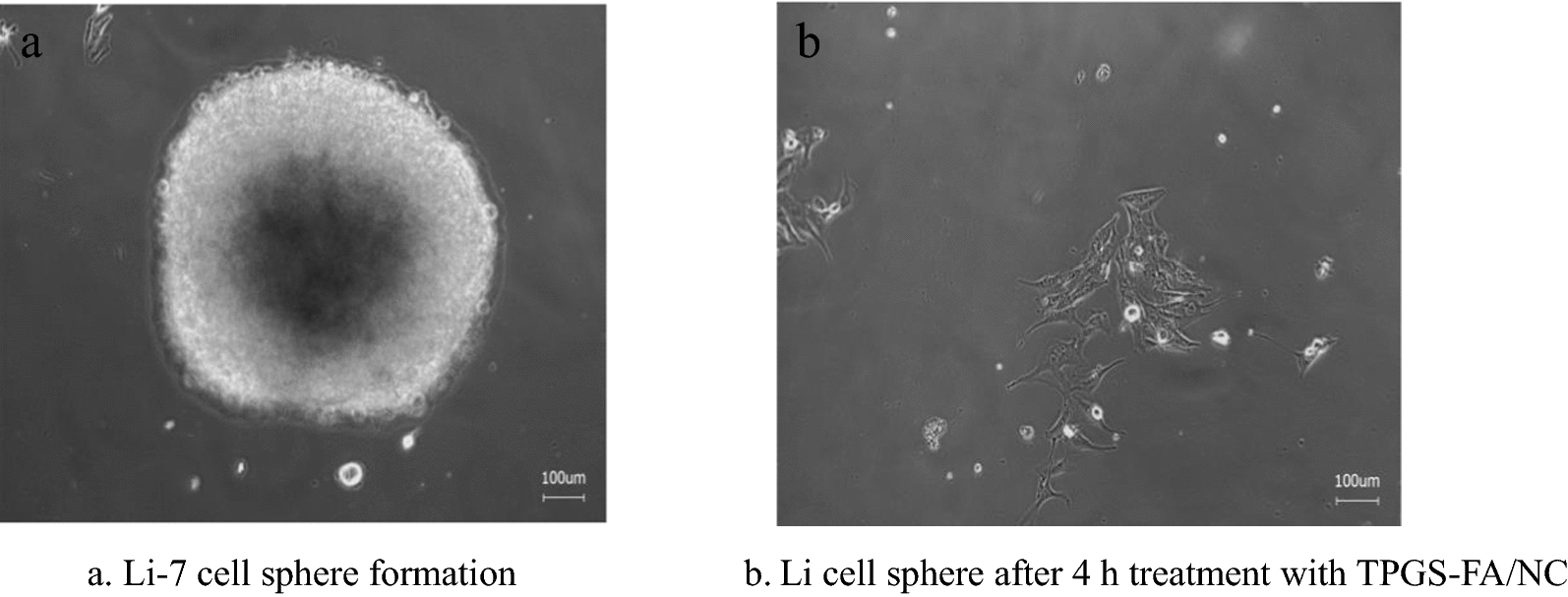
**a** Li-7 cell sphere formation **b** Li cell sphere after 4 h treatment with TPGS-FA/NC

We evaluated the capacity of TPGS-FA/NC to promote cytotoxicity in Li cells (Fig. 4). TPGS-FA/NC treatment suggested significantly better cytotoxicity than 5-Fu and NC. The induction of apoptosis by TPGS-FA/NC was further demonstrated by microscopic visualization of the morphology of Li-7 cells (Fig. 5) Notably, TPGS-FA/NC was able to remarkably suppress cell spheres growth compared to vehicle (Fig. 6).

### Ability of targeting TPGS-FA/NC to Li-7 Cells

**Fig. 7:**
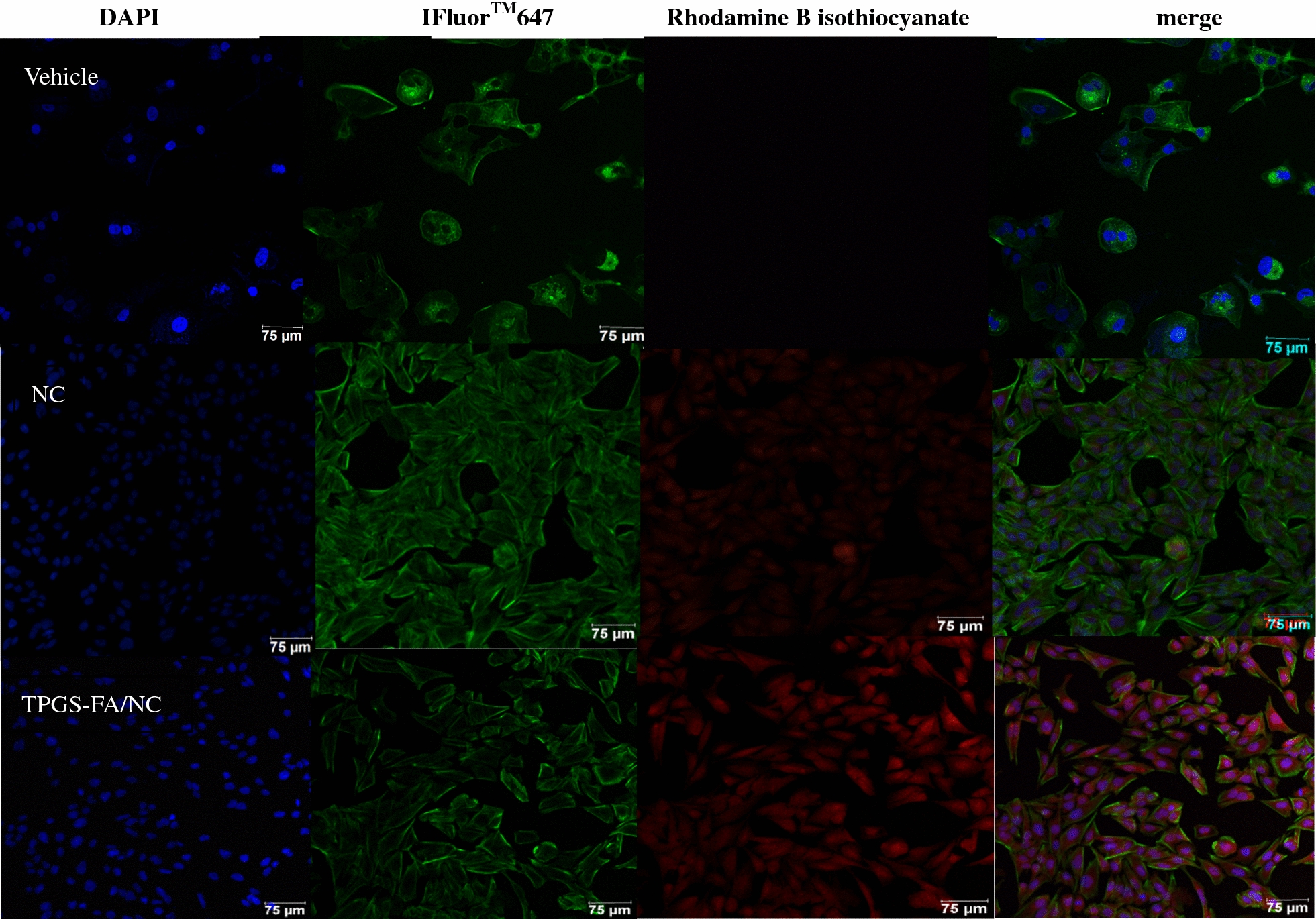
In vitro Li-7 cells binding of TPGS-FA/NC nanoparticles. Fluorescence staining of Li-7 cells, which were treated with TPGS-FA/NC and NC, was visualized by confocal microscopy. (blue: nucleus; green: cytoskeleton; red: TPGS-FA/NC nanoparticles. Scale bar: 75 μm for original images)

The Confocal microscopy imaging of the cells treated with Rhodamine B isothiocyanate-loaded nanoparticles showed that nanoparticles successfully entered the cell and then targeted to bind to cell nucleus. Our studies have shown an excellent targeted effect (Fig. 7).

### TPGS-FA/NC decreases ROS levels

**Fig. 8:**
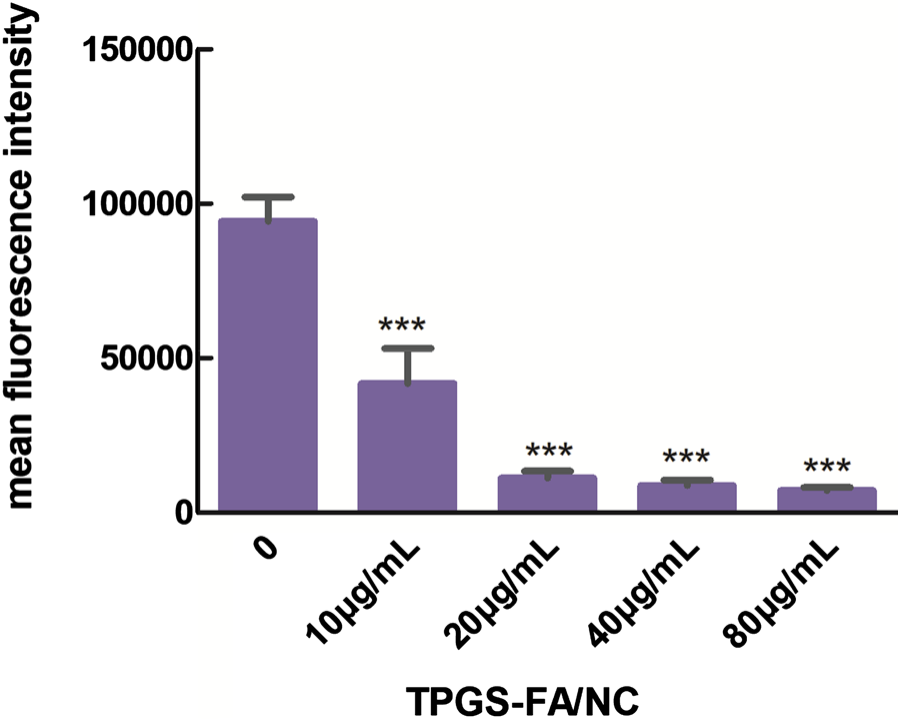
Bar graph plot of mean fluorescence intensity of Li-7 cell treated for 48 h with TPGS-FA/NC (0,10, 20, 40, or 80 μg/mL) (Data are mean ± SD n = 3, statistics were obtained using an unpaired t-test:*** P < 0.001)

We conducted a detection in Li-7 cells to compare the ROS levels of vehicle with a concentration gradient of TPGS-FA/NC (10, 20, 40, or 80 μg/mL). TPGS-FA/NC treatment significantly reduced the ROS levels, results confirmed TPGS-FA/NC accelerated cell inhibition (Table 1 and Fig. 8). Previous studies demonstrated ROS correlated with the tumor metastasis, invasion, and peritumor angiogenesis followed by the complex process of epithelial mesenchymal transformation (EMT) [13, 14]. These data here suggested that TPGS-FA/NC remarkably induced the cell apoptosis with decreased ROS levels.

**Table 1:** Mean fluorescence value of ROS levels

TPGS-FA/NC(μg/mL)	mean fluorescence intensity
0	97404
10	30266
20	13726
40	10440
80	7129

### TPGS-FA/NC suppresses protein expression levels

**Fig. 9:**
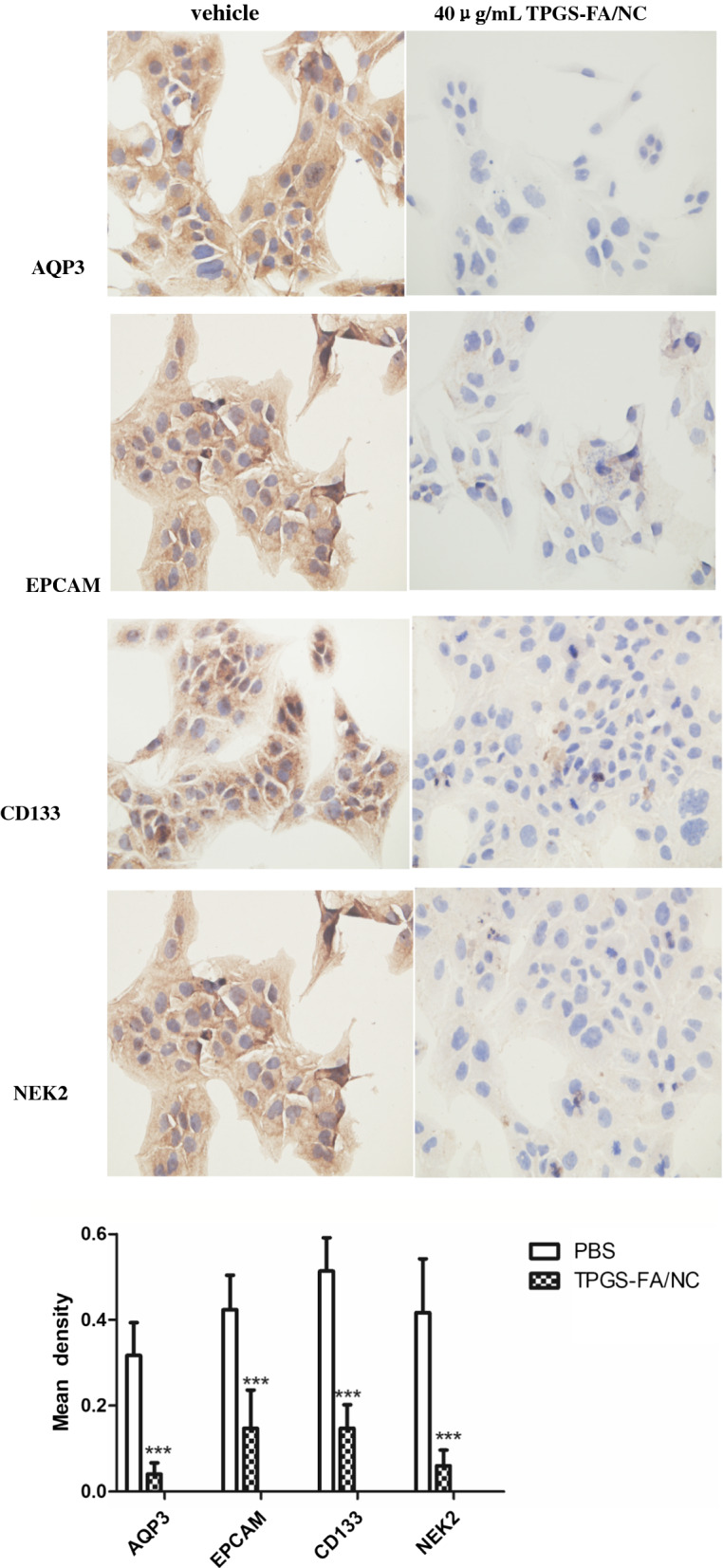
Detection of AQP3, NEK2,EPCAM, and CD133 (× 400) by IHC from Li cells. (Data are mean ± SD n = 3, statistics were obtained using an unpaired t-test: *** P < 0.001)

Frequently, CD133 is a well-known stem cell marker that has been demonstrated to promote the proliferation and invasion of liver cancer [15]. CD133^+^ hepatocellular carcinoma cells have showed high tumorigenic ability and strong self-renewal ability [16]. Therefore, abnormal expression of CD133 in tumor progression is significant in HCC [17]. AQP3 promotes the uptake of hydrogen peroxide and cell migration by regulating the Akt pathway. The high expression levels of AQP3 plays a carcinogenic role [18]. More importantly, Previous evidence has shown that upregulation of NEK2 expression play a key role in poor prognosis in HCC patients, indicating that NEK2 is a novel clinical biomarker for prediction of HCC and targeted therapies [19]. We examined the CD133, AQP3, EPCAM and NEK2 protein expression levels in vitro. Results suggested TPGS-FA/NC could suppress the NEK2, CD133, AQP3, and EPCAM expression levels in Li-7 cells by immunohistochemical staining analysis (Fig. 9), indicating that TPGS-FA/ NC was an excellent candidate drugs for cancer therapy.

## Conclusion

NEK2 protein and NC showed the strong binding interactions and tiny fluctuation with the transformation of constructions by molecular docking technology. Our findings provided pre-clinical proof-of-concept for the solid tumors treatment, which could provide a rationalized method in drug design for better therapeutic effect in liver cancer.

## Data Availability

Adequate and clear descriptions of the applied materials and software are provided in the materials and method section of manuscript. In addition, the obtained data is clearly justified by mentioning the figures and tables in the manuscript.
